# All that glitters is not gold: When motor and vocal tics in a child do not match Tourette syndrome: A case report

**DOI:** 10.1590/S1980-5764-2016DN1003014

**Published:** 2016

**Authors:** Raquel Quimas Molina da Costa, Rogério Paysano Marrocos, Marco Antonio Araujo Leite, Fabio Henrique Gobbi Porto

**Affiliations:** 1 MD, University of São Paulo Clinicas Hospital, Behavioral and Cognitive Neurology Unit, São Paulo, Brazil.; 2 MD, MSc, State of Rio de Janeiro Federal University (UNIRIO), Gaffree e Guinle University Hospital (HUGG), Rio de Janeiro, Brazil.; 3 MD, MSc, PhD, Movement Disorders Unit, Neurology Service, Department of Clinical Medicine, Antônio Pedro University Hospital (HUAP), Federal Fluminense University (UFF) Niterói, Brazil.; 4 MD, University of São Paulo Clinicas Hospital, Behavioral and Cognitive Neurology Unit, Department of Neurology and Cognitive Disorders Reference Centers (CEREDIC), São Paulo, Brazil.

**Keywords:** PKAN, Tourette syndrome, neurodegeneration, Tourettism, pantothenate kinase

## Abstract

The atypical form of Pantothenate Kinase-Associated Neurodegeneration (PKAN) tends to present at around the age of 14 years, has a heterogeneous presentation with extrapyramidal symptoms, and approximately one third of patients exhibit psychiatric problems. This paper reports the case of a patient with apparent typical symptoms of Tourette syndrome. However, the severity and poor response to treatment led to further investigation and the diagnosis of PKAN as a secondary cause of Tourettism was reached.

## RESUMO

A forma atípica de PKAN costuma se apresentar por volta dos 14 anos de idade, possui uma sintomatologia heterogênea, com sintomas extrapiramidais e, em cerca de um terço dos pacientes, também com a manifestação de sintomas psiquiátricos. O presente artigo relata o caso de uma paciente com sintomatologia típica da Síndrome de Tourette à primeira vista. Entretanto, a gravidade do quadro e pouca resposta ao tratamento levaram a uma maior investigação e ao diagnóstico de PKAN como causa secundária do *Tourettismo.*

## INTRODUCTION

Pantothenate Kinase-Associated Neurodegeneration (PKAN) belongs to a group of inherited neurodegenerative disorders characterized by neurodegeneration with brain iron accumulation (NBIA), especially in the basal ganglia.^1^ ^,^ ^2^ PKAN is an autosomal recessive disorder caused by a mutation in the PANK2 gene, in the chromosome 20p12.3-p13.^3^ The classical neuroradiological finding is known as the "eye of the tiger" sign, which is characterized by bilateral areas of T2-weighted MRI hyperintensity within a region of surrounding hypointensity in medial globus pallidi.^4^

There are two main phenotypes, defined according to age of onset. One is classical PKAN whose onset typically occurs before the age of 6 years presenting with gait or postural disturbances and developing extrapyramidal symptoms and corticospinal signs as the disease progresses. Most of these patients also have pigmentary retinopathy and are wheelchair bound within 15 years. Atypical PKAN usually presents around the age of 14 years, has less severe extrapyramidal impairment, slow progression, heterogeneous presentation, and may manifest neuropsychiatric symptoms in about 30% of cases.^2^ Early stages may present with depression, impulsivity and emotional outbursts, and in a few cases, Tourettism has been described as an early manifestation.^5^ ^,^ ^6^

Tourette syndrome (TS) is a neuropsychiatric disorder characterized by multiple motor and vocal tics lasting more than one year and childhood onset.^7^ In conditions where the frontal-striatal-thalamic-cortical (FSTC) circuitry is affected, such as tumors, long-term use of neuroleptics, head trauma, stroke, encephalitis and also with certain drugs, toxins, and post-infectious states, patients may present with Tourette-like phenomenology, a condition known as secondary TS, or Tourettism (TSM).^8^ ^-^ ^10^

Herein we described a case of atypical PKAN with Tourettism as the first presenting symptom. We discussed the neuroanatomical correlation of basal ganglia involvement in NBIA, and highlighted the importance of a complete investigation in atypical cases of TS.

## CASE REPORT

A 13-year-old girl was evaluated at the psychiatric service for motor and vocal tics. The symptoms had begun two years earlier and had progressed in intensity with time. She shook her hands in the air, hit her heels on the floor and smiled several times during speech. Her vocal tics were coughing, throat clearing and repeating several times the letter "A" as if stuttering. The patient also reported the sensation of "pain" and "jitters" before engaging the behavior that was relieved when performing the tics. Her past medical history included hyperactivity, without medical treatment. She had a normal birth without complications and her neuromotor development and school performance had always been within the normal range.

One year after the first evaluation, the tics intensified, hampering her school performance and social relationships. By this time, obscene tics and copropraxia such as masturbatory movements and clutching her genitals also emerged. 

**Figure 1:**
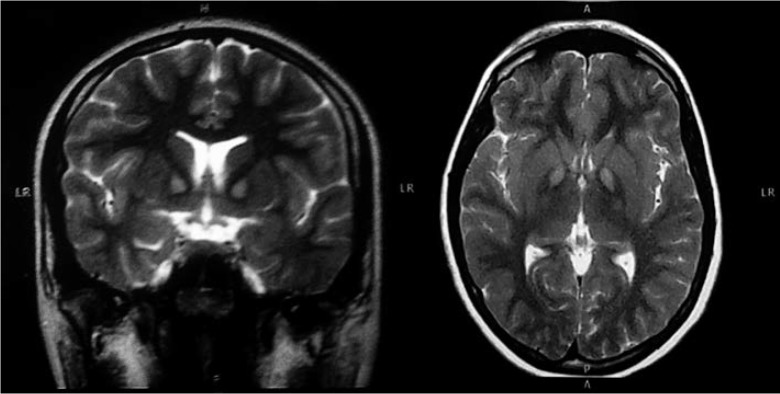
Coronal and axial T2-weighted MRI of patient A showing the "eye of the tiger sign".

She was initially treated with pimozide (a dopamine antagonist), with only partial response, but developed elevated prolactin levels and akathisia. Treatment was switched to risperidone 3 mg⁄day and topiramate, up to the dosage of 100 mg⁄day, was associated for improved control of tics and impulsivity. Rapid severity progression and poor response to treatment led to the hypothesis of a secondary cause of the tics and an MRI was ordered. A T2-weighted MRI showed bilateral areas of hyperintensity in the medial globus pallidi, a finding classically described as the "eye of the tiger" sign, and a typical indication of PKAN diagnosis (Figure 1). Genetic testing confirmed mutation in the PANK 2 gene and the diagnosis of PKAN. 

Ten months after the diagnosis, she developed choreic movements in lower and upper limbs, and athetosis in the right foot. Frequency of tics reduced despite no change in treatment dosage, and emotional control worsened, with frequent tantrums, aggression and more severe hyperactivity. School performance also declined, hindering school attendance. 

## DISCUSSION

We reported an atypical case of PKAN presenting with Tourettism. This patient showed apparent typical symptoms of TS, but severity and lack of therapeutic response to typical TS medication led to further investigation with radiologic imaging. The "eye of the tiger sign" is a specific MRI pattern, typically seen in neurodegeneration with brain iron accumulation (NBIA).^11^ PKAN accounts for 50% of NBIA cases and the presence of this sign is a key diagnostic feature of the disease, leading to genetic confirmation.^11^ A mutation analysis of PCR amplification in the coding region of the PANK 2 gene confirmed the patient's diagnosis.

Recent neuropathological and neuroimaging studies in patients with TS have shown both gain and loss of projection neurons in the globus pallidus and structural abnormalities within cortico-striato-pallido-thalamo-cortical pathways.^12^

Moreover, volumetric resonance imaging and post mortem studies of patients with TS have disclosed reduced levels of glutamate in the globus pallidus interna and altered hemispheric asymmetry of the putamen and globus pallidus.^12^ ^,^ ^13^ Thus, we hypothesized that dysfunction of basal ganglia circuitry caused by the iron accumulation may have promoted the manifestation of Tourette symptoms.

## CONCLUSION

This case highlights the broad spectrum of PKAN and the importance of this differential diagnosis in Tourettism, especially when severity and poor response to treatment is noted. A radiologic imaging study may provide an important key to diagnosis in such cases.
